# Relationship between Angiotensin II, Vascular Endothelial Growth Factor, and Arteriosclerosis Obliterans

**DOI:** 10.1155/2023/1316821

**Published:** 2023-02-21

**Authors:** Yulian Liu, Yuzhi Cui, Zongqi Zhou, Bin Liu, Zheng Liu, Gang Li

**Affiliations:** ^1^Department of Interventional Diagnosis and Treatment, Tai'an Traditional Chinese Medicine Hospital, Tai'an 271000, China; ^2^Department of Vascular Surgery, The Second Affiliated Hospital of Shandong First Medical University, Tai'an 271000, China; ^3^Department of Peripheral Vascular Diseases, Affiliated Hospital of Shandong University of Traditional Chinese Medicine, Jinan 250014, China

## Abstract

**Objective:**

To investigate the relationship between angiotensin II (Ang II), vascular endothelial growth factor (VEGF), and arteriosclerosis obliterans (ASO).

**Methods:**

60 ASO patients diagnosed and treated from October 2019 to December 2021 were selected for the observation group while 30 healthy physical examiners were for the control group. The general information (gender, age, history of smoking, diabetes, and hypertension) and arterial blood pressure (systolic and diastolic blood pressure) of the two groups were collected, and parameters like disease site and duration, Fontaine stage, and ankle-brachial index (ABI) of ASO patients have been evaluated. Ang II, VEGF, uric acid (UA), low-density lipoprotein (LDL), high-density lipoprotein (HDL), triglyceride (TG), and total cholesterol (TC) were also detected for the two groups. The variations in UA, LDL, HDL, TG, and TC among two groups along with levels of Ang II and VEGF in ASO patients in accordance to conditions like the general situation, disease duration, disease site, Fontaine stage, and ABI risk level have been studied to establish a correlation between Ang II and VEGF and ASO.

**Results:**

(1) The proportion of males with a history of smoking, diabetes, and hypertension was higher (*P* < 0.05) among ASO patients in comparison to the control group. The diastolic blood pressure, LDL, TC, Ang II, and VEGF levels were found to be higher (*P* < 0.05) whereas HDL was low (*P* < 0.01). (2) The level of Ang II in male patients with ASO was significantly higher than that in female ASO patients (*P* < 0.05). In ASO patients, the levels of Ang II and VEGF increased not only with age (*P* < 0.01) but also with progression in Fontaine stages II, III, and IV (*P* < 0.01). (3) Logistic regression analysis revealed Ang II and VEGF as risk factors for ASO. (4) An AUC (area under the ROC (receiver operator characteristic) curve) for Ang II and VEGF for the diagnosis of ASO was 0.764 (good) and 0.854 (very good), respectively, while their combined AUC in diagnosing ASO was 0.901 (excellent). The AUC of Ang II and VEGF together in diagnosing ASO was greater than that of Ang II and VEGF alone along with higher specificity as well (all *P* < 0.05).

**Conclusion:**

Ang II and VEGF were correlated with the occurrence and development of ASO. The AUC analysis demonstrates that Ang II and VEGF were highly discriminative of ASO.

## 1. Introduction

Arteriosclerosis obliterans (ASO) is a common peripheral arterial occlusive disease. It is manifested in systemic arteriosclerosis in the arteries of the lower limbs. In the early stages, the clinical manifestations involve limb cooling, fear of cold, numbness, intermittent claudication, etc. In the later stages, ulceration and gangrene may occur with amputation occurrence in serious cases [1]. ASO has been reported to involve more middle-aged and elderly people (over 45 years old) also with more males than females [2]. It is also reported that the incidence rate of ASO is as high as 15% ~20%, and the annual mortality is 4% ~6% [3]. As China's population ages, the incidence rate of ASO is increasing year by year affecting both physical and mental health as well as patient quality of life [4]. Atherosclerosis (AS) is the pathological basis of ASO [5] where the pathogenesis of AS is complex and there are many theories about it. Among them, the theory of chronic inflammation had played a crucial role in studying AS pathogenesis and also received widespread attention in the medical community [6, 7].

In 1997, researchers first found that Ang II can induce endothelial cells to produce adhesion molecules, which are closely related to inflammation. With the deepening of research, a new idea has been proposed that Ang II can cause inflammation and participate in the pathogenesis of AS [8].

Angiotensin II (Ang II) can induce the aggregation of a variety of inflammatory cytokines, lead to the proliferation of the middle layer of the artery, and promote inflammatory infiltration in the surrounding area of the middle layer of the artery, thus accelerating the development of AS. Its advantages in evaluation are increasingly prominent [9]. In addition, the abnormal proliferation of vascular smooth muscle cells (VSMCs) also plays an important role in the development of AS. As a critical functional factor of the renin-angiotensin-aldosterone system (RAAS), Ang II can act on VSMCs through autocrine and paracrine pathways, thus stimulating the abnormal proliferation of VSMCs [10] and stimulating angiogenesis in lower concentrations but inhibits in higher concentrations [11]. The process of angiogenesis involves the differentiation, proliferation, and migration of endothelial cells (ECs) and results in tubulogenesis and formation of vessels [12]. Ang II promotes EC proliferation and angiogenesis through the angiotensin type 1 receptor (AT1R) [13, 14]. Buharaliogl et al. found that Ang II-induced angiogenesis was phosphorylated by splenic tyrosine-mediated kinase through VEGF receptor-1.

Vascular endothelial growth factor (VEGF) is a highly specific mitogen of vascular endothelial cells [15] that can induce endothelial cell proliferation, increase vascular permeability, and promote inflammatory response [16]. Studies have found that once neovascularization occurs in AS plaques, the expression of VEGF and its receptor in AS plaques will increase, so it is inferred that there is a close association between VEGF expression and the development of AS plaques [17–20]. Simultaneously, studies have also confirmed [21] that VEGF has strong expression in smooth muscle and macrophages of atherosclerotic plaques. VEGF plays a crucial role in the formation of AS, which may be due to the fact that although VEGF can promote angiogenesis; these new blood vessels are often immature and prone to rupture, leading to thrombosis, and in-cooperated with highly expressed fibrinogen (FIB) to accelerate the formation and development of AS [22]. Meanwhile, studies have found that VEGF and its receptor are critically linked to the abnormal proliferation of VSMCs [23]. This study intends to explore the characteristics variations of Ang II and VEGF levels in ASO patients for preliminarily exploring the relationship between Ang II, VEGF, and ASO.

## 2. Material

Main experimental reagent and software product information.

## 3. Methods

### 3.1. Study Subjects

The subjects in the observation group were all ASO patients hospitalized in the peripheral vascular department of the Affiliated Hospital of Shandong University of Traditional Chinese Medicine from October 2019 to December 2021. The control groups were all from the health checkers in the physical examination center of the Affiliated Hospital at Shandong University of Traditional Chinese Medicine. This study was approved by the Ethics Committee of the Affiliated Hospital of Shandong University of Traditional Chinese Medicine ((2022) Lunlun-Review No. (108) -KY). The following data have been utilized as diagnostic criteria: ASO western medical diagnostic criteria [24] with (1) age > 40 years old; (2) history of smoking, diabetes, hypertension, hyperlipidemia, and other high-risk factors; (3) clinical manifestations of ASO in lower limbs; (4) arterial pulsation in the distal end of the ischemic limb weakened or disappeared; and (5) ABI ≤ 0.9; (6) color doppler ultrasound, CTA, MRA, DSA, and other imaging examinations showed stenosis or occlusion of the corresponding arteries. A clinical diagnosis of ASO can be made by meeting the following 4 diagnostic criteria: Fontaine staging standard [25] stage I: asymptomatic, stage II: intermittent claudication, stage III: resting pain, and stage IV: tissue ulcer and gangrene. The following case inclusion criteria were utilized: (1) those who met the diagnostic criteria of ASO, (2) 40 years old < age ≤ 80 years old, and (3) those who have signed the informed consent form. The following were case exclusion criteria: (1) those who have suffered from serious cardiovascular and cerebrovascular diseases in the past 3 months; (2) accompanied by serious infectious diseases, malignant tumors, and autoimmune diseases; (3) pregnant and lactating women; (4) those who have a mental illness or are unable to cooperate; and (5) incomplete data affecting judgment.

### 3.2. Study Subjects and Grouping

A total of sixty ASO patients who met the natriuretic excretion criteria were set as an observation group while 30 healthy physical examiners during the same period acted as a control group.

### 3.3. Clinical and Laboratory Variables

This represents the general data where the following clinical indicators were used: (1) gender, (2) age, (3) disease duration, (4) the site of the disease, (5) Fontaine staging, (6) smoking history, (7) history of diabetes, and (8) history of hypertension. The following parameters were utilized as laboratory variables for the detection of AS like (1) blood pressure: systolic blood pressure and diastolic blood pressure, (2) angiotensin II (Ang II), (3) vascular endothelial growth factor (VEGF), (4) ankle-brachial index (ABI), and (5) biochemical indicators: low-density lipoprotein (LDL), high-density lipoprotein (HDL), triglyceride (TG), total cholesterol (TC), and uric acid (UA).

### 3.4. Study Procedures

The following study procedures were utilized for this study that involves (1) measurement method of blood pressure: the subject rested calmly for 5 min before receiving the examination, took a sitting or lying position, used a mercury sphygmomanometer to measure three times, and calculated the average value of three times of blood pressure. The blood pressure of both arms was measured while the higher one was taken as the final blood pressure. (2) Measurement method of ABI: Nicolet vasoguard + channel blood vessel tester was used, and special personnel was assigned to detect it. The ABI of both sides was detected, and the lower was taken as the final value. (3) 3 ml of peripheral venous blood in fasting decubitus position was taken into a centrifuge after standing for 1 h at normal temperature, plasma was taken after centrifugation, and it was stored at -70°C for Ang II detection. Plasma Ang II was determined by radioimmunoassay. (4) 5 ml of fasting peripheral venous blood was taken and placed in the coagulation-promoting tube, centrifuged at 4000 r/min for 5 min, the upper serum was taken for examination, and serum VEGF was measured with enzyme-linked immunosorbent assay. (5) 2 ml of fasting peripheral venous blood was taken, and LDL, HDL, TG, TC, and UA were detected by AU5800 automatic biochemical analyzer (model: 5821, no. DSH-001).

### 3.5. Statistical Analysis

The statistical software IBM SPSS28.0 has been utilized for data processing. All measured data were expressed as mean ± standard deviation (*x* ± *s*). *T*-test was used for the comparison of data conforming to normal distribution and homogeneity of variance, and analysis of variance was used for the comparison among multiple groups. Nonparametric test was used for data not conforming to normal distribution. The count data were expressed by frequency and composition ratio (*n* (%)). We have utilized the *X*^2^ test, a binary logistic regression model for correlation analysis; the ROC curves for the diagnostic value of the detection index and the AUC of ROC is compared by Medcalc software. The *P* values (*P* < 0.05) were statistically considerable.

## 4. Results

### 4.1. Subject Demographics and Study Variables

In the 60 cases in the observation group, around 42 were males, and 18 females with an average age of 60.57 ± 6.16 years were found. The shortest disease period was of 1 year while the longest of 28 years with an average duration of 5.47 ± 6.00 years. There were 13 cases located in the left lower limb, 26 in the right lower limb, and 21 in both lower limbs. According to the Fontaine stage, there were 14, 7, and 39 cases in stages II, III, and IV, respectively. Of the 30 cases in the control group, there were 11 males and 19 females with an average age of 58.07 ± 9.38 years.

**Figure 1:**
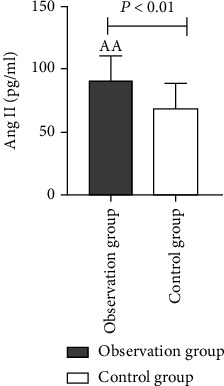
Comparison of Ang II levels between the two groups.

**Figure 2:**
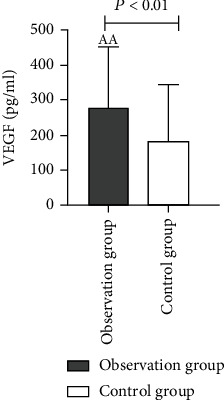
Comparison of VEGF levels between the two groups.

As compared to the control group, a higher proportion of males with a history of smoking as well as diabetes were found in the observation group and also the levels of diastolic blood pressure, TC, Ang II (Figure 1), and VEGF (Figure 2) were higher while the HDL level was lower. These variations among the two groups were statistically considerable with *P* values (*P* < 0.01). In comparison to the control group, the proportion of hypertension history and LDL level in the observation group was higher; also, these differences between the two groups were statistically considerable with *P* values (*P* < 0.05). We have not found any statistically considerable differences in age, systolic blood pressure, TG, and UA between the two groups (*P* > 0.05) (Table 1).

### 4.2. Comparisons within Stratified Subgroups of Cases

A total of sixty ASO patients were stratified according to gender, age, duration, and site of disease, diabetes and hypertension history, Fontaine stage, and ABI risk grade; and the changes in Ang II and VEGF levels were observed. We have found the following results for Ang II and VEGF comparison with different parameters.

### 4.3. Comparison of Ang II and VEGF Levels between Different Genders of ASO Patients

**Figure 3:**
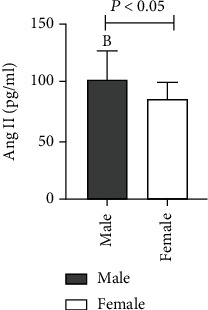
Comparison of Ang II levels in ASO patients with different genders.

**Figure 4:**
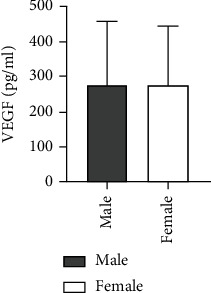
Comparison of VEGF levels in ASO patients of different genders.

The level of Ang II was found higher in males than females for 60 cases of ASO, and the difference between the two groups was statistically significant (*t* = −2.542, *P* = 0.019). The VEGF level was also found higher in males than females but without any statistically considerable difference between the two groups (*Z* = −0.355, *P* = 0.723) (Table 2 and Figures 3 and 4).

### 4.4. Comparison of Ang II and VEGF Levels in ASO Patients at Different Ages

**Figure 5:**
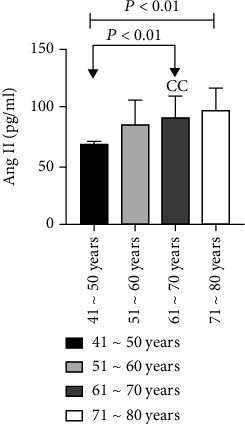
Comparison of Ang II levels among ASO patients of different ages.

**Figure 6:**
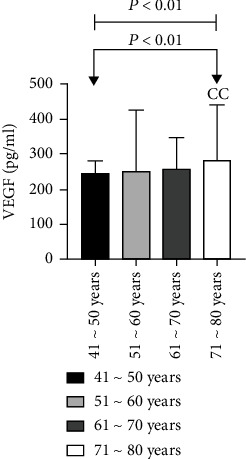
Comparison of VEGF levels in ASO patients of different ages.

60 patients with ASO were divided into groups according to the age interval of 10 years. There were 3 patients aged 41 to 50 years, 23 patients aged 51 to 60 years, 31 patients aged 61 to 70 years, and 3 patients aged 71 to 80 years. We have found that, with an increase in age, the levels of Ang II and VEGF also increased in ASO patients, and the difference was statistically significant as analyzed by the Kruskal-Wallis *H* test (*P* < 0.01). Further, the level of Ang II in the 61-70 years old age group was significantly higher than that in the 41-50 years old group (*P* < 0.01), and there was not any statistically considerable difference between the other two groups (*P* > 0.05). Also, the VEGF level in the 71-80 years old group was found higher than that of the 41-50 years old group (*P* < 0.01) while there was not any statistically considerable difference between the other two groups (*P* > 0.05) (Table 3 and Figures 5 and 6).

### 4.5. Comparison of Ang II as well as VEGF Levels among Patients with ASO at Different Sites

**Figure 7:**
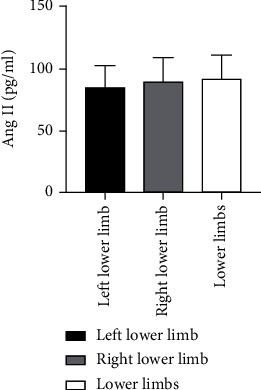
Comparison of Ang II levels among different sites of ASO patients.

**Figure 8:**
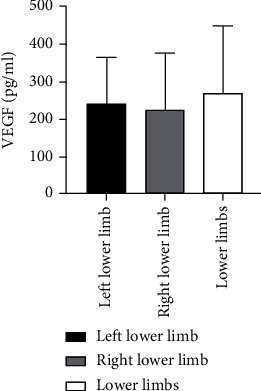
Comparison of VEGF levels in ASO patients with different disease sites.

Sixty patients with ASO were divided into three groups according to the different disease sites including 13, 26, and 21 cases at the left lower limb, right lower limb, and both lower limbs, respectively. Kruskal-Wallis *H* test showed that there was not any statistically considerable difference for Ang II as well as VEGF levels among these groups (*P* > 0.05) (Table 4 and Figures 7 and 8).

### 4.6. Comparison of Ang II as well as VEGF Levels among ASO Patients with Different Disease Durations

**Figure 9:**
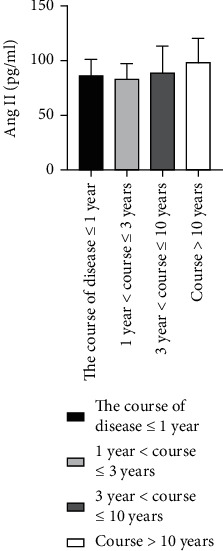
Comparison of Ang II levels in ASO patients with different disease duration groups.

**Figure 10:**
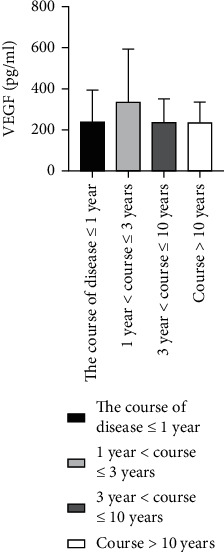
Comparison of VEGF levels in ASO patients with different disease duration groups.

We grouped 60 patients with ASO according to the disease duration [26], where 29 patients had a duration of disease ≤ 1 year, 4 cases with 1 year < duration ≤ 3 years, 18 cases with 3 years < duration ≤ 10 years, and 9 cases with disease duration > 10 years. The Kruskal-Wallis *H* test showed that there was not any statistically considerable difference for Ang II as well as VEGF levels among these groups (*P* > 0.05) (Table 5 and Figures 9 and 10).

### 4.7. Comparison of Ang II as well as VEGF Levels among Patients of ASO with or without a Smoking History

**Figure 11:**
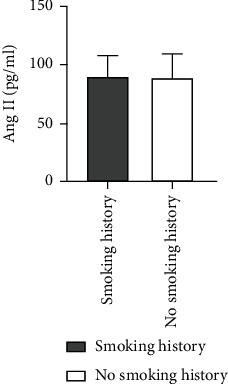
Comparison of Ang II level between ASO patients with and without smoking history.

**Figure 12:**
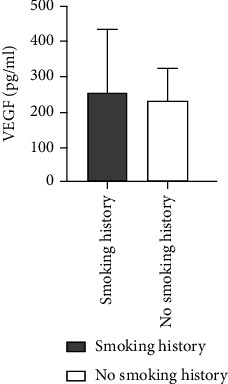
Comparison of VEGF levels between ASO patients with and without smoking history.

The level of Ang II in ASO patients with smoking history was higher than that in patients without smoking history, and there was not any statistically considerable difference between the two groups (*Z* = −0.333, *P* = 0.739). Also, higher VEGF levels were found among patients of ASO with smoking history in comparison to patients without smoking history with no statistically considerable difference between the two groups (*Z* = −0.287, *P* = 0.774) (Table 6 and Figures 11 and 12).

### 4.8. Comparison of Ang II as well as VEGF Levels among ASO Patients with or without a History of Diabetes

**Figure 13:**
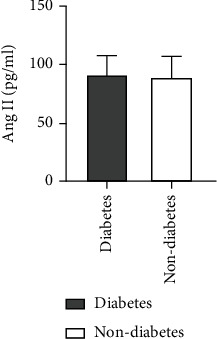
Comparison of Ang II level between ASO patients with and without diabetes history.

**Figure 14:**
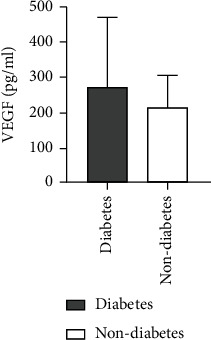
Comparison of VEGF levels between ASO patients with and without diabetes history.

A higher level of Ang II was found in patients of ASO with a history of diabetes in comparison to those without any diabetes history. There was not any statistically considerable difference between the two groups (*Z* = −0.096, *P* = 0.923). Also, a higher VEGF level was observed for ASO patients with a history of diabetes in comparison to those without any diabetes history. There was not any statistically considerable difference between the two groups (*Z* = −0.836, *P* = 0.403) (see Table 7 and Figures 13 and 14).

### 4.9. Comparison of Ang II as well as VEGF Levels among ASO Patients with or without Hypertension

**Figure 15:**
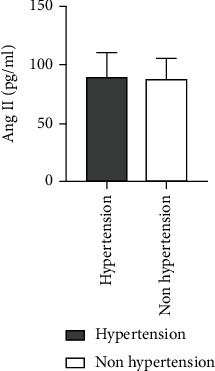
Comparison of Ang II level between ASO patients with and without hypertension history.

**Figure 16:**
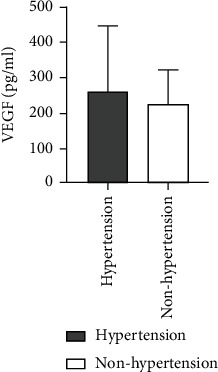
Comparison of VEGF levels between ASO patients with and without hypertension history.

A higher level of Ang II was observed for ASO patients with hypertension history in comparison to those without hypertension, and there was not any statistically considerable difference between the two groups (*Z* = −0.952, *P* = 0.341). Also, a higher VEGF level was found for ASO patients with a hypertension history in comparison to those without a history of hypertension. There was not any statistically considerable difference between the two groups (*Z* = −0.247, *P* = 0.805) (Table 8 and Figures 15 and 16).

### 4.10. Comparison of Ang II as well as VEGF Levels among ASO Patients with Different Fontaine Stages

**Figure 17:**
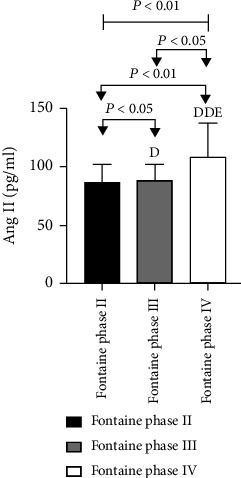
Comparison of Ang II levels in ASO patients with different clinical stages.

**Figure 18:**
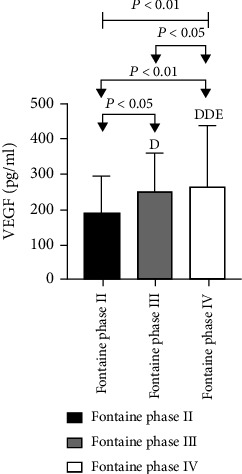
Comparison of VEGF levels in ASO patients with different clinical stages.

In 60 patients with ASO, the Ang II, as well as VEGF levels among different Fontaine stages, was statistically significant as analyzed by the Kruskal-Wallis *H* test (*P* < 0.01). Among them, a higher level of Ang II and VEGF was observed for patients with Fontaine III in comparison to patients with Fontaine II with a statistically considerable difference among these groups (*P* < 0.05). Also, significantly higher levels of Ang II and VEGF were observed for patients with Fontaine IV in comparison to patients with Fontaine II with a statistically considerable difference among these (*P* < 0.01). Further, significantly higher levels of Ang II and VEGF were found in the Fontaine IV group in comparison to the Fontaine III group (*P* < 0.05) (Table 9 and Figures 17 and 18).

### 4.11. Comparison of Ang II as well as VEGF Levels among ASO Patients at Different ABI Risk Levels

**Figure 19:**
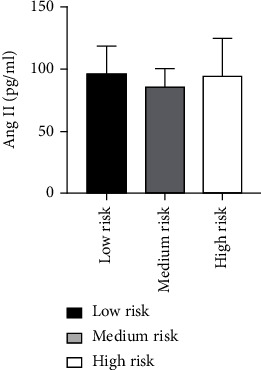
Comparison of Ang II levels among ASO patients with different risk grades.

**Figure 20:**
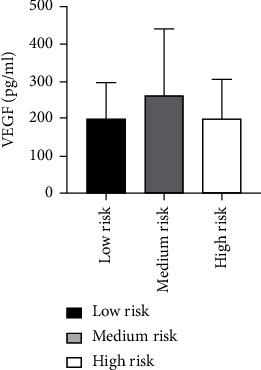
Comparison of VEGF levels in ASO patients with different risk grades.

According to the ABI of patients, the risk grade was divided [27]: the low-risk group (0.7 ≤ ABI < 0.9, *n* = 13), the medium-risk group (0.4 ≤ ABI < 0.7, *n* = 41), and the high-risk group (ABI < 0.4, *n* = 6). The levels of Ang II in 60 ASO patients with different risk grades were not statistically significant (*P* = 0.147) as analyzed by ANOVA. Also, there was not any statistically considerable difference in VEGF levels among 60 ASO patients with different risk grades as analyzed by the Kruskal-Wallis *H* test (*P* = 0.487) (Table 10 and Figures 19 and 20).

### 4.12. Logistic Regression Analysis of Ang II, VEGF, and ASO

The correlation between Ang II, VEGF, and ASO was statistically analyzed. Taking ASO patients as dependent variables while Ang II and VEGF as independent variables, a binary logistic regression analysis was conducted. The results showed that Ang II (partial regression coefficient = 0.038, OR = 1.216, 95% confidence interval (CI): 0.997-1.343, *P* value = 0.006) and VEGF (partial regression coefficient = 0.059, OR = 1.744, 95% CI: 1.076-1.115, *P* = 0.017) were positively correlated with ASO. These results indicate that Ang II and VEGF could be regarded as risk factors for ASO (Table 11).

### 4.13. Receiver Operating Curve Analysis

**Figure 21:**
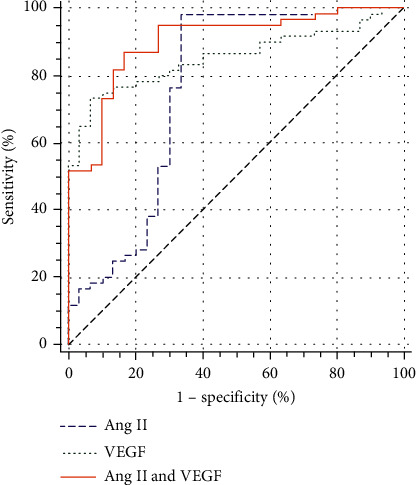
Area under the ROC curve.

The results from this study reveal that the AUC of Ang II and VEGF in diagnosing ASO was 0.764 (good) and 0.854 (very good), respectively, indicating their value as moderate in diagnosing ASO. On other hand, the AUC of the combined diagnosis of Ang II and VEGF was 0.901 (excellent), indicating that the combined diagnosis of Ang II and VEGF has a higher diagnostic value for ASO. Medcalc software was used to compare the AUC of Ang II, VEGF, and their combined diagnosis. We observed a statistically considerable difference between the two groups (*P* < 0.05). Among them, the AUC of Ang II and VEGF combined diagnosis was the highest, followed by VEGF and then Ang II with the lowest value (Tables 12 and 13 and Figure 21).

## 5. Discussion

Atherosclerosis (AS) is the pathological basis of ASO [5], and its pathogenesis is very complex and involves a variety of theories. Among these, the theory of chronic inflammation plays a crucial role in AS pathogenesis and has received widespread attention in the medical community [7]. As a chronic inflammatory disease, AS has the characteristics of classic inflammatory proliferation, degeneration, and exudation. Previous studies mentioned the occurrence and development of AS to be closely related to chronic inflammatory reactions [28–31]. Ang II can promote inflammatory response by inducing the aggregation of a variety of inflammatory cytokines [32]. High level of angiotensin II is one of the typical features of atherosclerosis patients. A large number of studies have confirmed that angiotensin II can effectively activate the Notch1 signaling pathway in a variety of cells. This study also confirmed the effective activation of Notch1 signaling pathway [33], and the overexpression of Notch1 receptor and ligand can stimulate the accumulated macrophages to show an M1-type transformation and release a large number of proinflammatory mediators such as IL-1*β* and TNF-*α*. Induce and enhance the inflammatory response of the body, and downregulate the Th2 inflammatory cytokines that inhibit the inflammatory response, such as IL-6, to further promote the inflammatory response [34]. Also, as reported in a previous study, intraplaque angiogenesis plays a critical role in developing AS. VEGF is an important proangiogenic factor found to be involved in regulating and inducing angiogenesis among plaques [35]. Dunmore et al. [36] found that VEGF overexpression existed in unstable plaques, and its content was significantly positively correlated with plaque instability, suggesting that the mismatch between VEGF overexpression and other related factors in plaques may be an important reason for the immaturity of new blood vessels. In addition, changes in the structure or function of VSMCs are the main pathological basis of AS [37, 38] where VSMC proliferation is closely linked to AS development. Ang II is a powerful promoter of VSMC phenotype transformation and plays an important role in inducing VSMC proliferation and migration as well as the progression of AS plaque formation [39, 40]. Dong et al. [41] found that Ang II promoted the secretion of endothelin 1 by vascular endothelial cells, which in turn promoted the production of Ang II by vascular endothelial cells, and the two cooperated to promote the proliferation of VSMC. The possible mechanism was that Ang II, as an autocrine growth factor, could directly induce the mRNA expression of c-myc and fos, thus causing the proliferation of VSMC. Wang et al. [42] also found that Ang II could promote abnormal proliferation of VSMC in rats with spontaneous hypertension, and the AT1R blocker captopril could inhibit the proliferation. In addition, studies have found that VEGF and its receptor are closely related to the proliferation of VSMCs [23]. Therefore, Ang II and VEGF can stimulate an inflammatory response, induce angiogenesis in plaques, and participate in VSMC proliferation and migration with the possibility of promoting the occurrence and development of AS. In another study, Zhou et al. [43] found that the effect of Ang II on AS may be independent of its effect on blood pressure, which is basically consistent with the conclusions of relevant animal studies. It was pointed out that Ang II may also be crucially associated with human AS. In recent studies, CRP has been shown to be directly involved in the formation of AS plaques. During an inflammatory reaction of AS tube wall, Peng and coworkers found that Ang II could induce the production of CRP in VSMCs [44]. Recent studies have also confirmed [45] that Ang II can induce the expression of CRP in macrophages through a variety of signaling pathways, thereby playing a proinflammatory role. Simultaneously, studies have confirmed [20] that if the expression of VEGF and its receptor in AS plaques is significantly increased; then, it indicates new blood vessel formation in AS plaques, depicting a crucial association of VEGF with AS.

In this study, the level of Ang II in ASO patients was considerably higher in comparison to the control group (*P* < 0.01), which indicates an increase in Ang II level being related to ASO. The mechanism for ASO induction on increasing Ang II level may be linked to induction of inflammatory reaction and the stimulation of VSMC proliferation. Ang II can promote inflammatory response by inducing a variety of inflammatory cytokines to aggregate and activate NF-*κ*B [22]. Excessive Ang II secretion stimulates an abnormal proliferation of VSMCs in blood vessels, activates relevant signaling pathways, promotes the abnormal elevation of inflammatory factors such as interleukin-6 (IL-6) and nitric oxide (NO), causes damage to blood vessels, and then promotes the occurrence of AS [46]. The binary logistic regression analysis showed Ang II to be a risk factor for ASO. The AUC of Ang II was 0.764 (good), which indicates that Ang II has a moderate diagnostic value for ASO, with a sensitivity of 98.3% and a specificity of 66.7%. It can also be seen that Ang II is an effective index to evaluate the severity of ASO disease while having a certain reference value for the diagnosis of ASO. Simultaneously, the results of this study reveal that the VEGF level of ASO patients was considerably higher in comparison to the control group (*P* < 0.01), indicating that the increase in VEGF level is related to ASO. The mechanism by which the elevated VEGF level promotes the occurrence of ASO may be related to the induction of angiogenesis in AS plaques and the promotion of inflammatory response [22]. Among them, VEGF and its tyrosine kinase receptor pathway make endothelial cells proliferate and migrate, increase vascular permeability, and participate in regulating and inducing angiogenesis. However, in the initial stage, the increase in VEGF level stimulates the abnormal proliferation of vascular endothelial cells, resulting in immature neovascularization [20]. Studies have shown that immature and pathological neovascularization mostly exists in AS unstable plaques [47]. Binary logistic regression analysis showed that Ang II and VEGF were risk factors for ASO. The AUCs of Ang II and VEGF in the diagnosis of ASO were 0.764 (good) and 0.854 (very good), respectively, indicating their moderate diagnostic value for ASO. They have a certain correlation with the occurrence and development of ASO and also reflect the severity of ASO to a certain extent. The AUC was compared with Medcalc software, and it was found that the AUC of Ang II and VEGF together in the diagnosis of ASO was 0.901 (excellent), which was higher than the AUC of these two indicators alone. This clearly indicates that the combined detection of the two indicators is more valuable in the diagnosis of ASO.

In conclusion, Ang II and VEGF were correlated with the occurrence and development of ASO. At the same time, the AUC analysis demonstrates that Ang II and VEGF were highly discriminative of ASO. However, the limitations of the study, due to time constraints, a small sample size, and only established cases, have been analyzed. Hence, a more rigorous, multicenter, larger sample, double-blind study needs to be designed for further analysis.

## Figures and Tables

**Table 1:** Clinical data from the observation group compared to the control group.

	Observation group (*n* = 60)	Control group (*n* = 30)	*P* value
Gender (%)			
Male	42 (70.00)	11 (36.67)	0.002
Female	18 (30.00)	19 (63.33)	
Smoking history (%)			
Yes	27 (45.00)	3 (10.00)	0.001
No	33 (55.00)	27 (90.00)	
History of diabetes (%)			
Yes	36 (60.00)	5 (16.67)	≤0.001
No	24 (40.00)	25 (83.33)	
History of hypertension (%)			
Yes	35 (58.33)	9 (30.00)	0.011
No	25 (41.67)	21 (70.00)	
Blood pressure (mmHg)			
Systolic blood pressure	141.80 ± 19.29	136.33 ± 20.20	0.215
Diastolic blood pressure	88.10 ± 10.25^aa^	80.30 ± 12.60	0.004
Age (years)	60.57 ± 6.16	58.07 ± 9.38	0.171
LDL (mmol/L)	3.03 ± 0.93^a^	2.62 ± 0.86	0.040
HDL (mmol/L)	0.94 ± 0.22^aa^	1.18 ± 0.34	≤0.001
TG (mmol/L)	1.77 ± 0.77	1.16 ± 0.43	0.764
TC (mmol/L)	4.83 ± 1.10^aa^	4.12 ± 1.04	0.004
UA (*μ*mol/L)	326.08 ± 98.41	347.33 ± 81.46	0.311
Ang II (pg/ml)	90.66 ± 19.69^aa^	68.80 ± 20.02	≤0.001
VEGF (pg/ml)	277.7 ± 171.60^aa^	182.08 ± 162.40	≤0.001

Note: compared with the control group, ^a^*P* < 0.05, and ^aa^*P* < 0.01.

**Table 2:** Comparison of Ang II and VEGF levels between different genders of ASO patients.

Group	*n*	Ang II (pg/ml)	VEGF (pg/ml)
Male	42	101.98 ± 25.22^b^	278.22 ± 175.80
Female	18	85.81 ± 14.63	276.51 ± 166.32
*T* value/*Z* value		-2.542	-0.355
*P* value		0.019	0.723

Note: compared with females, ^b^*P* < 0.05.

**Table 3:** Comparison of Ang II and VEGF levels in ASO patients at different ages.

Group	*n*	Ang II (pg/ml)	VEGF (pg/ml)
41~50 years	3	68.36 ± 3.92	243.52 ± 38.78
51~60 years	23	85.18 ± 20.98	251.34 ± 174.94
61~70 years	31	91.76 ± 18.25^cc^	256.09 ± 90.97
71~80 years	3	97.58 ± 19.28	282.45 ± 158.58^cc^
*P* value		0.006	0.001

Note: compared with the 41-50-year-old group, ^cc^*P* < 0.01.

**Table 4:** Comparison of Ang II as well as VEGF levels among patients with ASO at different sites.

Group	*n*	Ang II (pg/ml)	VEGF (pg/ml)
Left lower limb	13	84.65 ± 19.02	242.34 ± 124.67
Right lower limb	26	89.13 ± 19.40	226.22 ± 152.74
Lower limbs	21	90.19 ± 20.11	267.15 ± 183.91
*P* value		0.426	0.549

**Table 5:** Comparison of Ang II as well as VEGF levels among ASO patients with different disease durations.

Group	*N*	Ang II (pg/ml)	VEGF (pg/ml)
The duration of disease ≤ 1 year	29	86.25 ± 14.83	238.91 ± 155.20
1 year < duration ≤ 3 years	4	83.02 ± 14.28	334.99 ± 258.89
3 years < duration ≤ 10 years	18	88.61 ± 24.64	236.52 ± 114.25
Duration > 10 years	9	98.30 ± 21.99	235.21 ± 100.08
*P* value		0.222	0.912

**Table 6:** Comparison of Ang II as well as VEGF levels among ASO patients with or without a smoking history.

Group	*n*	Ang II (pg/ml)	VEGF (pg/ml)
Smoking history	21	88.94 ± 18.63	252.49 ± 183.46
No smoking history	39	87.83 ± 21.07	228.34 ± 94.56
*Z* value		-0.333	-0.287
*P* value		0.739	0.774

**Table 7:** Comparison of Ang II as well as VEGF levels among ASO patients with or without diabetes history.

Group	*n*	Ang II (pg/ml)	VEGF (pg/ml)
Diabetes	31	89.07 ± 19.24	273.03 ± 197.49
Nondiabetes	29	87.96 ± 19.80	213.05 ± 92.91
*Z* value		-0.096	-0.836
*P* value		0.923	0.403

**Table 8:** Comparison of Ang II as well as VEGF levels among ASO patients with or without hypertension.

Group	*n*	Ang II (pg/ml)	VEGF (pg/ml)
Hypertension	35	90.42 ± 19.45	259.10 ± 188.27
Nonhypertension	25	85.93 ± 19.29	222.95 ± 100.32
*Z* value		-0.952	-0.247
*P* value		0.341	0.805

**Table 9:** Comparison of Ang II as well as VEGF levels among ASO patients at different clinical stages.

Group	*n*	Ang II (pg/ml)	VEGF (pg/ml)
Fontaine phase II	14	85.39 ± 17.00	189.21 ± 106.46
Fontaine phase III	7	87.72 ± 14.32^d^	249.68 ± 109.87^d^
Fontaine phase IV	39	107.81 ± 29.98^dde^	260.92 ± 177.04^dde^
*P* value		0.007	0.001

Note: compared with Fontaine phase II, ^d^*P* < 0.05, and ^dd^*P* < 0.01. Compared with Fontaine III, ^e^*P* < 0.05.

**Table 10:** Comparison of Ang II as well as VEGF levels among ASO patients with different risk grades.

Group	*N*	Ang II (pg/ml)	VEGF (pg/ml)
Low risk (0.7 ≤ ABI ≤ 0.9)	13	96.47 ± 22.11	200.83 ± 96.06
Medium risk (0.4 ≤ ABI < 0.7)	41	85.25 ± 15.32	263.95 ± 176.87
High risk (ABI < 0.4)	6	93.98 ± 30.00	201.57 ± 102.29
*P* value		0.147	0.487

**Table 11:** Correlation analysis of Ang II, VEGF, and ASO.

Factor	*r*	SE	Wald value	*P*	OR	95% CI
Ang II	0.038	0.068	6.823	0.006	1.216	0.997-1.343
VEGF	0.059	0.015	5.248	0.017	1.744	1.076-1.115

**Table 12:** Area under ROC curve.

Factor	AUC	SE	*P*	95% CI
Ang II	0.764	0.065	≤0.001	0.663-0.847
VEGF	0.854	0.039	≤0.001	0.764-0.920
Ang II and VEGF	0.901	0.034	≤0.001	0.835-0.967

**Table 13:** Comparison of area under ROC curve of each index.

Factor	AUC	SE	*Z* values	*P*	95% CI
Ang II vs. VEGF	0.090	0.072	1.257	0.020	0.651-0.859
Ang II vs. Ang II + VEGF	0.137	0.051	2.675	0.007	0.710-0.952
VEGF vs. Ang II + VEGF	0.047	0.027	1.725	0.028	0.820-0.954

**Table 14:** Main experimental reagent.

The name of the reagent	Source of reagents
Ang II	Shenzhen New Industry Biomedical Engineering Co., Ltd.
VEGF	Beijing Jianping Jinxing Biotechnology Co., Ltd.

**Table 15:** Main software product information.

The name of the software	Source software
IBMSPAA28.0	IBM company
Medcalc	Beijing Huanzhong Ruechi Technology Co., Ltd.

## Data Availability

The data presented in the study may be made available from the corresponding author upon reasonable request.
